# Pitavastatin Is a Highly Potent Inhibitor of T-Cell Proliferation

**DOI:** 10.3390/ph14080727

**Published:** 2021-07-27

**Authors:** Linda Voss, Karina Guttek, Annika Reddig, Annegret Reinhold, Martin Voss, Luca Simeoni, Burkhart Schraven, Dirk Reinhold

**Affiliations:** 1 Institute of Molecular and Clinical Immunology, Otto-von-Guericke-University Magdeburg, 39120 Magdeburg, Germany; linda.voss@med.ovgu.de (L.V.); karina.guttek@med.ovgu.de (K.G.); annika.reddig@med.ovgu.de (A.R.); annegret.reinhold@med.ovgu.de (A.R.); martin.voss@med.ovgu.de (M.V.); luca.simeoni@med.ovgu.de (L.S.); burkhart.schraven@med.ovgu.de (B.S.); 2 Health Campus Immunology, Infection and Inflammation (GC-I3), Medical Fakulty, Otto-von-Guericke-University Magdeburg, 39120 Magdeburg, Germany

**Keywords:** drug repositioning, Pitavastatin, inhibitor of T-cell proliferation, ERK1/2 activation, apoptosis

## Abstract

Repositioning of approved drugs is an alternative time- and cost-saving strategy to classical drug development. Statins are 3-hydroxy-3-methylglutaryl-CoA (HMG CoA) reductase inhibitors that are usually used as cholesterol-lowering medication, and they also exhibit anti-inflammatory effects. In the present study, we observed that the addition of Pitavastatin at nanomolar concentrations inhibits the proliferation of CD3/CD28 antibody-stimulated human T cells of healthy donors in a dose-dependent fashion. The 50% inhibition of proliferation (IC50) were 3.6 and 48.5 nM for freshly stimulated and pre-activated T cells, respectively. In addition, Pitavastatin suppressed the IL-10 and IL-17 production of stimulated T cells. Mechanistically, we found that treatment of T cells with doses <1 µM of Pitavastatin induced hyperphosphorylation of ERK1/2, and activation of caspase-9, -3 and -7, thus leading to apoptosis. Mevalonic acid, cholesterol and the MEK1/2 inhibitor U0126 reversed this Pitavastatin-mediated ERK1/2 activation and apoptosis of T cells. In summary, our results suggest that Pitavastatin is a highly potent inhibitor of T-cell proliferation, which induces apoptosis via pro-apoptotic ERK1/2 activation, thus representing a potential repositioning candidate for the treatment of T-cell-mediated autoimmune diseases.

## 1. Introduction

Pharmaceutical drug development is a time- and cost-intensive process. Usually, it takes 10 to 15 years from compound discovery to approval of a new drug and costs around US$1.3 billion to US$1.7 billion [1,2,3]. About 50% of drugs that reach phase III of clinical testing fail to obtain approval [4]. An alternative way to de novo drug discovery is repurposing of established drugs for new indications [5]. Financial and development risks are reduced because repositioning candidates have already passed several phases of clinical trials, and their pharmacokinetic profiles and safety are well described [6]. Therefore, the time frame for drug development can be shortened, because most of the preclinical testing, safety assessment and, in some cases, drug-formulation development have already been completed [7].

Epidemiological studies have shown that 3–5% of the population are affected by autoimmune diseases [8]. There are nearly 100 distinct autoimmune disorders described, including organ-specific ones, such as type I diabetes, as well as systemic autoimmune diseases involving multiple organs, such as systemic lupus erythematosus (SLE) [9]. Autoimmune diseases arise from a combination of genetic and environmental factors, which lead to defective regulation of lymphocyte activation and stimulation of self-reactive T lymphocytes that have escaped control mechanisms [10]. Different T-cell subsets are involved in disease progression, including regulatory T cells, which usually inhibit disease development by tightly controlling autoreactive T-cell and B-cell responses and naïve T cells, which may undergo clonal expansion and activation upon exposure to self-antigens [11]. These activated autoreactive T cells are of particular importance in chronic autoimmune inflammation [12]. 

Previously, our group demonstrated that commonly used immunosuppressive drugs, such as cyclosporin A and dexamethasone, fail to suppress the proliferation of pre-activated T cells [13]. Thus, the strategy to look for substances having an effect on T cells at different stages of activation may contribute to improved therapeutic approaches against autoimmune diseases. 

Recently, we performed a screening of 786 FDA-approved drugs with the aim to find compounds that inhibit proliferation of both, freshly stimulated and pre-activated peripheral blood mononuclear cells (PBMCs). In addition to Adefovir Dipivoxil, already described [14], we identified Pitavastatin in this screening as a potent drug that inhibited cell proliferation in a nanomolar dose range under both experimental culture conditions.

Pitavastatin as a member of the statin drug family is widely used for the treatment of hypercholesterolemia [15]. Statins are competitive inhibitors of 3-hydroxy-3-methylglutaryl-CoA (HMG-CoA) reductase, a rate-limiting enzyme of cholesterol biosynthesis that catalyzes the conversion of HMG-CoA to mevalonate (MVA) [16]. MVA is a precursor not only for cholesterol synthesis but also for the synthesis of isoprenoids, i.e., geranygeranyl pyrophosphate (GGPP) and farnesyl pyrophosphate (FPP) [17]. These isoprenoids are important for post-translational prenylation of signaling molecules such as GTP-binding proteins, thus facilitating their anchoring to the cell membrane [18]. 

Some signaling molecules require membrane association to mediate their cellular functions, including cell survival, proliferation, differentiation and cytoskeletal organization [19]. In addition to lipid-lowering effects, animal experiments and clinical studies have shown that statins exhibit anti-inflammatory and immunomodulatory effects [20]. The effects of statins on the immune system are pleiotropic and include inhibition of T-cell activation, proliferation and migration [21,22]. It was shown that simvastatin and lovastatin reduce the expression of lymphocyte function-associated antigen 1 (LFA-1), necessary for leucocyte endothelial cell adhesion [23]. Statins inhibit Interferon-γ (IFN-γ)-induced major histocompatibility complex (MHC) class II expression by blocking class II transactivator (CIITA) gene expression, thereby leading to impaired antigen presentation [24]. Furthermore, statins promote shifting of the T-cell response from a pro-inflammatory Th1 into an anti-inflammatory Th2 profile in experimental autoimmune encephalomyelitis (EAE), the animal model of multiple sclerosis [25]. 

Here, we show that Pitavastatin at nanomolar concentrations dose-dependently inhibits the proliferation of stimulated human T cells. Mechanistically, Pitavastatin induced hyperphosphorylation of ERK1/2, and activation of the caspase-9 and -3/7, leading to apoptosis. Our results suggest that Pitavastatin is a highly potent inhibitor of T-cell proliferation, and a potential repositioning candidate for treatment of T-cell-mediated autoimmune diseases.

## 2. Results

### 2.1. Pitavastatin Suppresses Proliferation, as Well as IL-10 and IL-17 Production of Freshly Stimulated T Cells, but Shows No Effect on T-Cell Activation 

**Figure 1:**
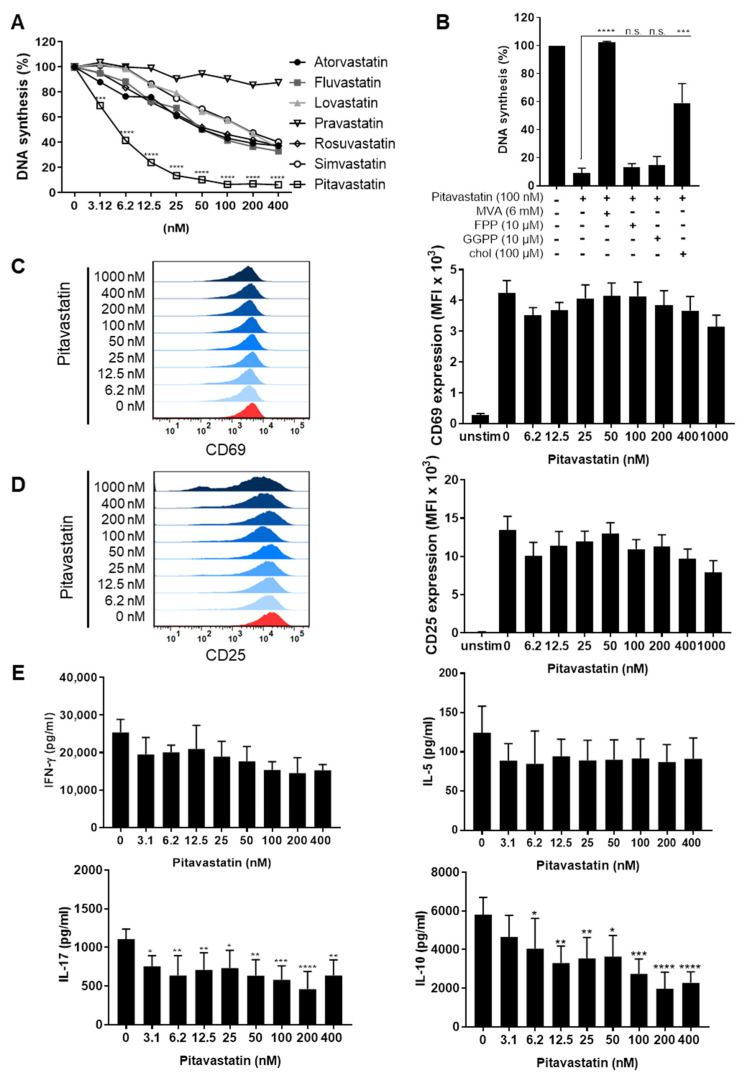
Pitavastatin inhibited proliferation, as well as IL-10 and IL-17 production of stimulated T cells, but had no influence on T-cell activation. Human resting T cells freshly stimulated with anti-CD3/CD28 antibodies were cultured with increasing concentrations of different statins (**A**) or 100 nM Pitavastatin in the presence or absence of either 6 mM MVA, 10 µM FPP, 10 µM GGPP or 100 µM cholesterol (chol) (**B**) for 72 h. DNA synthesis was determined by standard [^3^H]-thymidine uptake. [^3^H]-TdR incorporation is shown as mean percentage ± SEM of DNA synthesis in relation to control cultures (vehicle) set to 100%. (**C**–**E**) Human T cells were freshly stimulated with anti-CD3/CD28 antibodies and incubated with increasing concentrations of Pitavastatin. Expression levels of the T-cell activation markers CD69 after 16 h (**C**) and CD25 after 48 h (**D**) were analyzed by flow cytometry. Representative histograms (left) and mean fluorescence intensity (MFI, right) are displayed. Cell-culture supernatants were harvested 72 h after treatment, and concentrations of IFN-γ, IL-5, IL-17 and IL-10 were determined with specific ELISA (**E**). Data are presented as the mean ± SEM of *n* = 4 independent experiments. Statistical analysis was performed with One-Way ANOVA and Dunnett’s Multiple Comparison Analysis Test as post hoc test. (**** *p* ≤ 0.0001, *** *p*  ≤  0.001, ** *p*  ≤  0.01, * *p*  ≤  0.05).

We studied the effects of multiple statins on human T-cell proliferation by measuring [3H]-thymidine ([^3^H]-TdR) incorporation. T cells were freshly stimulated with anti-CD3/CD28 antibodies and incubated for 72 h with Atorvastatin, Fluvastatin, Lovastatin, Pravastatin, Rosuvastatin, Simvastatin and Pitavastatin, at concentrations ranging from 3.12 to 400 nM. All statins, except Pravastatin, significantly inhibited T-cell proliferation (Figure 1A). Unexpectedly, the most potent compound was Pitavastatin, with a half maximal inhibitory concentration (IC50) value of 3.6 nM, followed by Fluvastatin (76 nM) and Atorvastatin (79 nM). 

Due to these results, Pitavastatin was selected for further characterization. Inhibition of HMG-CoA reductase by statins not only leads to cholesterol depletion but also to reduction of several intermediate products of the mevalonate pathway, such as MVA, FPP or GGPP. To determine whether and to which extent supplementation of MVA, FPP, GGPP or cholesterol restores the proliferation of Pitavastatin-treated T cells, cell cultures were incubated with 100 nM Pitavastatin, together with 6 mM MVA, 10 µM FPP, 10 µM GGPP or 100 µM cholesterol. Interestingly, the Pitavastatin-dependent inhibition of T-cell proliferation was completely restored by the addition of MVA and partially by the addition of cholesterol. FPP and GGPP could not overcome the inhibitory effect of Pitavastatin (Figure 1B). This result suggests that cholesterol synthesis rather than protein prenylation might be responsible for the observed suppression of proliferation. 

To further investigate whether Pitavastatin has a direct effect on T-cell activation, T cells were stimulated with anti-CD3/CD28 antibodies and incubated with increasing concentrations of Pitavastatin. The expression of the activation markers CD69 and CD25 was assessed after 16 and 48 h, respectively. As expected, stimulation of T cells with anti-CD3/CD28 antibodies alone induced the expression of both activation markers. Conversely to the effect on T-cell proliferation, treatment with Pitavastatin did not significantly alter the expression of CD69 and CD25 (Figure 1C,D). Similarly, we did not detect any significant changes in IFN-γ and IL-5 levels in cell-culture supernatants. In contrast, the concentrations of IL-10 and IL-17 were found to be significantly reduced in Pitavastatin-treated T-cell cultures 72 h after T-cell stimulation (Figure 1E). Together, these results clearly show that T-cell proliferation, as well as the production of IL-10 and IL-17, can be efficiently suppressed by Pitavastatin at low nanomolar concentrations.

### 2.2. Pitavastatin Induces Apoptosis in Freshly Stimulated T Cells at Low Concentrations

**Figure 2:**
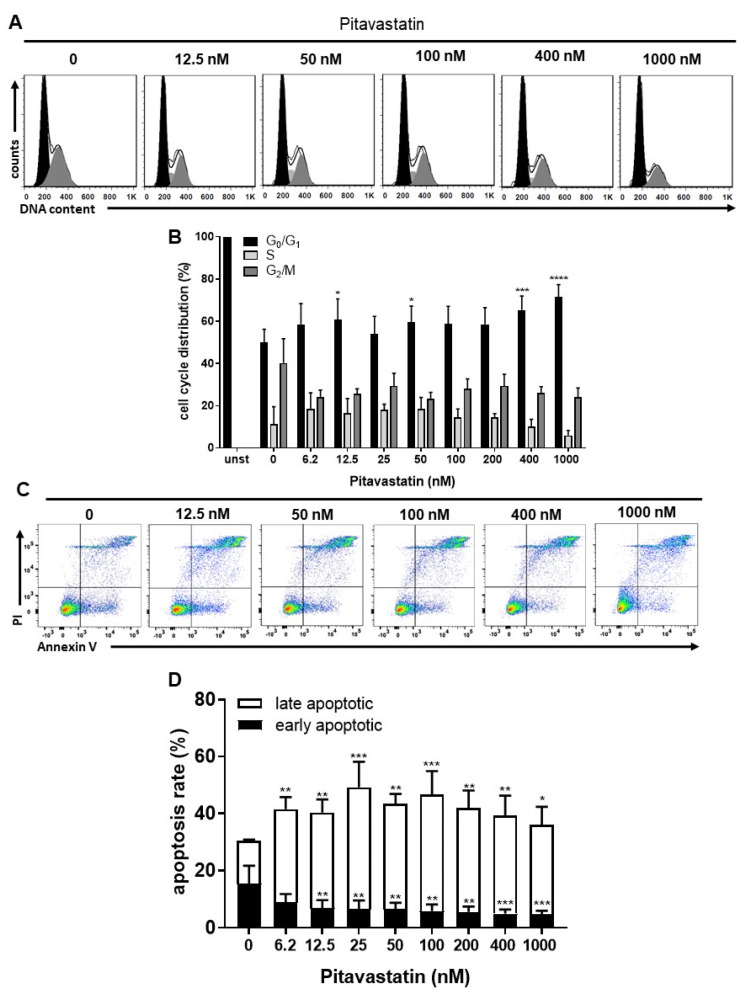
Pitavastatin blocked cell-cycle progression and induced cell death in freshly stimulated T cells. Human T cells were freshly stimulated with anti-CD3/CD28 antibodies and cultured with increasing concentrations of Pitavastatin for 72 h. DNA content and cell-cycle phase of cells were determined by using propidium iodide staining and flow cytometric analysis. Representative histograms of DNA content are shown in (**A**) (G_0_/G_1_ (black), S (light gray) and G_2_/M (dark gray)). (**B**) The bars indicate the percentage of cells in G_0_/G_1_ phase, S phase and G_2_/M phase. Data are presented as the mean + SEM of n = 4 independent experiments. Cells were stained after 72 h with Annexin V-FITC/PI for flow cytometric analysis. (**C**) Representative flow cytometry dot plots and (**D**) quantification of early and late apoptotic cells of *n* = 4 independent experiments are shown. Statistical analysis was performed with One-Way ANOVA and Dunnett’s Multiple Comparison Analysis Test as post hoc test. (**** *p* ≤ 0.0001, *** *p * ≤  0.001, ** *p*  ≤  0.01, * *p*  ≤  0.05).

We next asked the question whether the diminished proliferation of Pitavastatin-treated T cells is due to cell-cycle arrest in the G_0_/G_1_ phase or to apoptosis. We investigated the effect of Pitavastatin on cell-cycle progression of stimulated T cells after incubation for 72 h, using flow cytometry. As shown in Figure 2A,B, the percentage of T cells in the G_0_/G_1_ phase increased from 50 ± 6% in control T-cell cultures to 72 ± 6% in cell cultures treated with 1000 nM Pitavastatin. Conversely, the percentage of cells in the G_2_/M phase decreased from 40 ± 12% in control cultures to 24 ± 5% in treated cell cultures. This effect was found to be significant at Pitavastatin concentrations above 400 nM. These findings suggest that high concentrations of Pitavastatin induce cell-cycle arrest at the G_0_/G_1_ phase.

To investigate, whether this Pitavastatin-induced cell cycle arrest is associated with the induction of apoptosis, freshly stimulated T cells were incubated with increasing concentrations of Pitavastatin for 72 h and subsequently stained with Annexin V-FITC/propidium iodide (PI) for flow cytometric analysis. As presented in Figure 2C,D, the percentage of apoptotic cells significantly increased already to 41.5 ± 4.0% after incubation with the lowest Pitavastatin dose applied compared to the control cultures (30.4 ± 5.4%).

**Figure 3:**
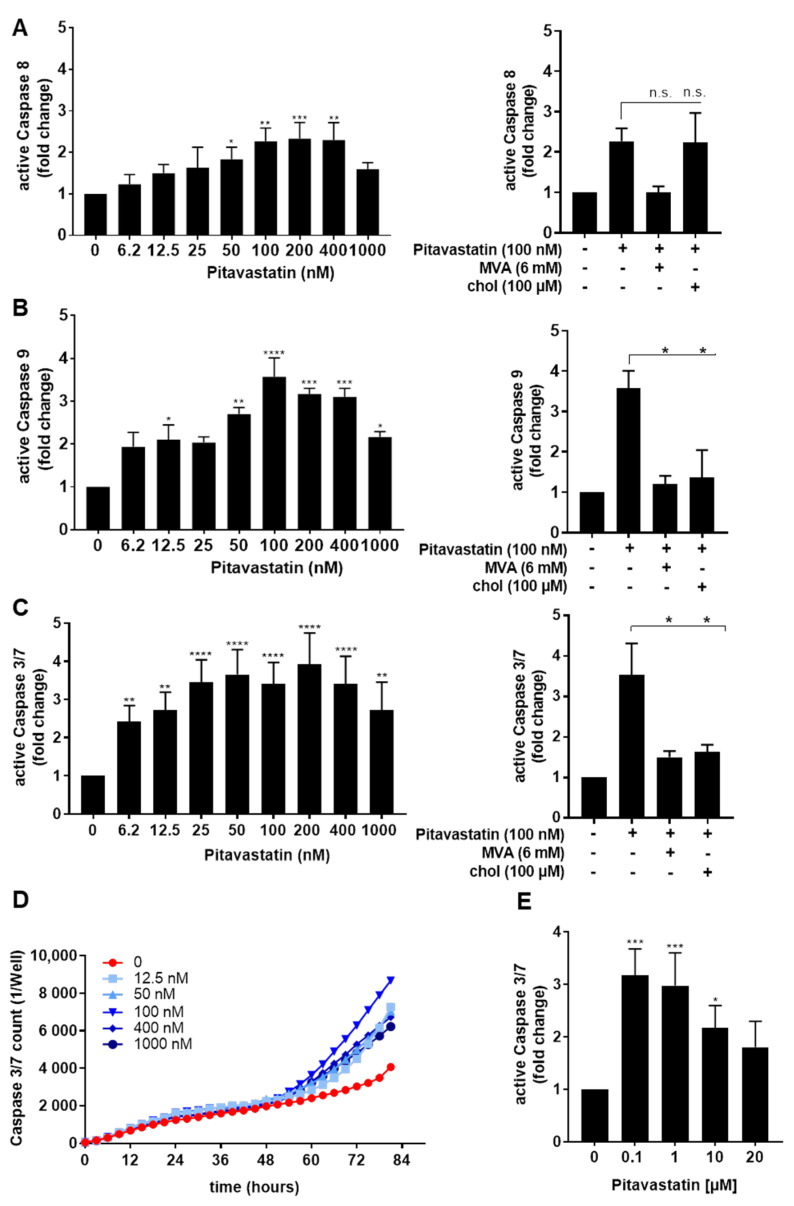
Pitavastatin activated caspases in freshly stimulated T cells. Resting human T cells were freshly stimulated with anti-CD3/CD28 antibodies and cultured with increasing concentrations of Pitavastatin or 100 nM Pitavastatin in the presence or absence of 6 mM MVA or 100 µM cholesterol (chol). Cells were stained after 72 h with active caspase-8-binding FITC-IETD-FMK (**A**), active caspase 9-binding FITC-LEHD-FMK reagent (**B**) or CellEvent™ Caspase-3/7 Green detection reagent (**C**) for flow cytometric analysis. Mean + SEM of relative caspase expression of *n* = 3 independent experiments are displayed. (**D**) For kinetic analyses, human T cells were stimulated with anti-CD3/CD28 antibodies and cultured with increasing concentrations of Pitavastatin in the presence of Caspase-3/7 Green detection reagent. Kinetic measures of the number of caspase-3/7 positive cells were recorded by the IncuCyte S3 imaging system at 3 h intervals for 84 h. (**E**) Resting human T cells were freshly stimulated with anti-CD3/CD28 antibodies and cultured with high concentrations of Pitavastatin. Cells were stained after 72 h with CellEvent™ Caspase-3/7 Green detection reagent. Results of *n* = 3 independent experiments are presented as mean values of relative caspase expression. Statistical analysis was performed with One-Way ANOVA and Dunnett’s Multiple Comparison Analysis Test as post hoc test. (**** *p* ≤ 0.0001, *** *p*  ≤  0.001, ** *p*  ≤  0.01, * *p * ≤  0.05).

To further examine the role of caspases in Pitavastatin-induced apoptosis, the levels of active caspase-3/7, caspase-8 and caspase-9 were measured in T cells treated with different concentrations of Pitavastatin for 72 h (Figure 3A–C). Caspase-8 initiates death-receptor-induced apoptosis, whereas caspase-9 acts through mitochondrial damage. These caspases subsequently cleave downstream caspases, such as caspase-3 or -7, leading to cell death. We found that Pitavastatin induced the activation of all three caspases. Flow cytometric analyses showed that treatment with 100 nM Pitavastatin for 72 h enhanced the expression of active caspase-8 up to 2.2-fold (Figure 3A left). When T cells were co-incubated with 100 nM Pitavastatin together with MVA or cholesterol, MVA could completely prevent caspase-8 activation, whereas cholesterol showed no effect (Figure 3A, right). As shown in Figure 3B, the expression of active caspase-9 was enhanced up to 3.5-fold in the presence of Pitavastatin, at a concentration of 100 nM, compared to untreated control T cells. Supplementation of MVA or cholesterol downregulated the expression of active caspase-9 to the level observed in control cells (Figure 3B, right). The expression of the active caspases-3/7 was enhanced up to 3.4-fold in the presence of Pitavastatin at a concentration of 100 nM compared to control T cells (Figure 3C, left). Supplementation of MVA or cholesterol reverse expression of active caspase-3/7 to the degree observed in control cells (Figure 3C, right). It was noticeable that a further increase in Pitavastatin concentration (1000 nM) attenuated the expression of all active caspases measured (Figure 3A–C, left).

To investigate the kinetics of Pitavastatin-induced apoptosis, we next monitored the signal of a specific green fluorescent dye for caspase-3/7 enzyme activity by IncuCyteS3^®^ live cell imaging over a period of 82 h (Figure 3D). Pitavastatin induced an increase in caspase-3/7 activity after 50 h, indicating a delayed apoptotic mechanism. Here, we observed the highest caspase-3/7 induction at a concentration of 100 nM Pitavastatin, whereas higher concentrations attenuated the caspase expression. To further validate this effect, we treated cells with increasing Pitavastatin concentrations up to 20 µM. We noticed that the caspase-3/7 expression was highest at the lowest Pitavastatin concentration of 100 nM (Figure 3E). Taken together, these data suggest that Pitavastatin induced apoptosis, especially at lower concentrations. 

### 2.3. Pitavastatin Induces Strong ERK1/2 Phosphorylation at Low Concentrations

**Figure 4:**
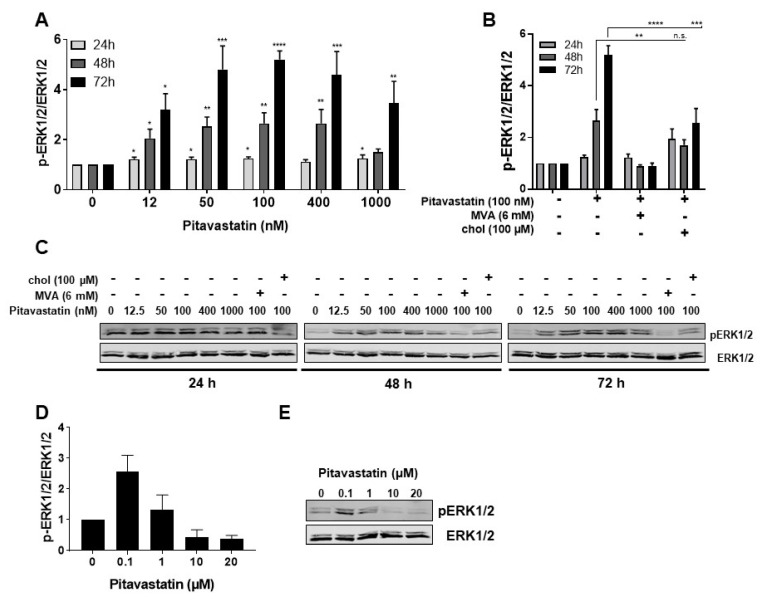
Pitavastatin enhanced ERK phosphorylation in freshly stimulated T cells. Resting human T cells were freshly stimulated with anti-CD3/CD28 antibodies and cultured with increasing concentrations of Pitavastatin (**A**) or 100 nM Pitavastatin in the presence or absence of either 6 mM MVA or 100 µM cholesterol (chol). (**B**) After 24 h, 48 h or 72 h incubation, cells were lysed and analyzed by Western blot. (**A**,**B**) Relative expression of p-ERK1/2 based on densitometric quantification. Quantitative data are presented as mean + SEM from *n* = 4 independent experiments. (**C**) Representative Western blot images of p-ERK1/2 and total ERK1/2. (**D**,**E**) Resting human T cells were freshly stimulated with anti-CD3/CD28 antibodies and cultured with high concentrations of Pitavastatin. After 48 h of incubation, cells were lysed and analyzed by Western blot. (**D**) Relative expression of p-ERK1/2 based on densitometric quantification. Quantitative data are presented as the mean + SEM from *n* = 3 independent experiments. (**E**) Representative Western blot images of p-ERK1/2 and total ERK1/2. Statistical analysis was performed with One-Way ANOVA and Dunnett’s Multiple Comparison Analysis Test as post hoc test. (**** *p* ≤ 0.0001, *** *p  *≤  0.001, ** *p*  ≤  0.01, * *p*  ≤  0.05).

To elucidate the mechanism of Pitavastatin-induced apoptosis, we investigated the potential role of MAPK signaling. The expression and activation of ERK1/2 were measured by Western blot after exposure of human freshly stimulated T cells to Pitavastatin over a period of 24 to 72 h (Figure 4A,C). Interestingly, we detected a strong phosphorylation of ERK1/2 in the presence of Pitavastatin. As shown in Figure 4A, ERK1/2 phosphorylation was already slightly increased after 24 h incubation (1.25-fold at 100 nM Pitavastatin) and even more clearly detectable after 48 h. Highest ERK1/2 phosphorylation was observed after 72 h at a concentration of 100 nM (5.2-fold). When cells were co-incubated with Pitavastatin and MVA, the ERK1/2 phosphorylation was entirely reversed. Addition of cholesterol led to a partial reduction of ERK1/2 phosphorylation (Figure 4B). These results were surprising, because several reports described the opposite phenomenon of reduced ERK1/2 phosphorylation in different cell systems [26,27,28]. To compare our experimental setting with data published in the previous reports applying much higher statin concentrations, we incubated freshly stimulated T cells with 100 nM Pitavastin, as well as with concentrations of 10 and 20 µM. ERK1/2 phosphorylation was analyzed after 48 h (Figure 4D,E). As expected, the ERK1/2 phosphorylation was increased after incubation of T cells with 100 nM Pitavastatin. Indeed, we observed that, in T cells incubated with high Pitavastatin concentrations, the ERK1/2 phosphorylation was reduced (0.3-fold at 20 µM Pitavastatin). Taken together, these data show that Pitavastatin induces ERK hyperphosphorylation at low nanomolar concentrations and suppresses ERK phosphorylation at concentrations of 10 µM and higher.

### 2.4. ERK1/2 Hyperphosphorylation Mediates Pitavastatin-Induced Apoptosis

**Figure 5:**
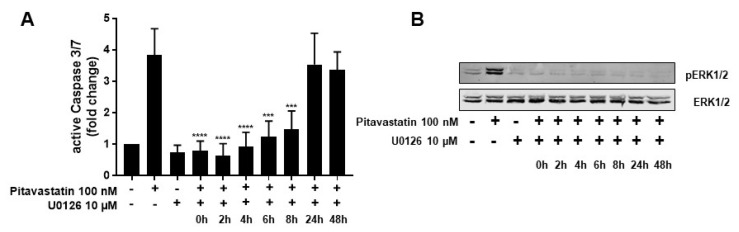
ERK hyperphosphorylation is responsible for Pitavastatin-induced apoptosis. Resting human T cells were freshly stimulated with anti-CD3/CD28 antibodies and cultured with 100 nM Pitavastatin in the presence or absence of 10 µM of the MEK1/2 inhibitor U0126. (**A**) Cells were stained after 72 h with CellEvent™ Caspase-3/7 Green detection reagent. Mean + SEM of relative caspase expression of *n* = 3 independent experiments are displayed. (**B**) Representative Western blot images of p-ERK1/2 and total ERK1/2. Statistical analysis was performed with One-Way ANOVA and Dunnett’s Multiple Comparison Analysis Test as post hoc test. (**** *p* ≤ 0.0001, *** *p*  ≤  0.001).

To determine whether the strong phosphorylation of ERK is responsible for the observed increased apoptosis in Pitavastatin-treated T cells, we used the specific MEK1/2 inhibitor U0126 to prevent ERK1/2 phosphorylation. Therefore, T cells were stimulated with CD3/CD28 antibodies and incubated with 100 nM Pitavastatin. The MEK inhibitor was added at different time points upon stimulation (0, 2, 4, 6, 8, 24 and 48 h), and the phosphorylation of ERK1/2 and active caspase-3/7 were analyzed 72 h upon stimulation. As shown in Figure 5A, treatment with Pitavastatin for 72 h induced caspase-3/7, as expected. This effect was clearly reduced when U0126 was added at early time points up to 8 h. Treatment with U0126 alone showed no effect on caspase-3/7 expression. The addition of the inhibitor 24 or 48 h after stimulation did not prevent induction of apoptosis. As shown in Figure 5B, phosphorylation of ERK1/2 was effectively prevented by U0126 at all timepoints. These results suggest that low doses of Pitavastatin induce apoptosis through sustained activation of pro-apoptotic ERK.

### 2.5. Pitavastatin Suppresses Proliferation and Induced Apoptosis in Pre-Activated T Cells

**Figure 6:**
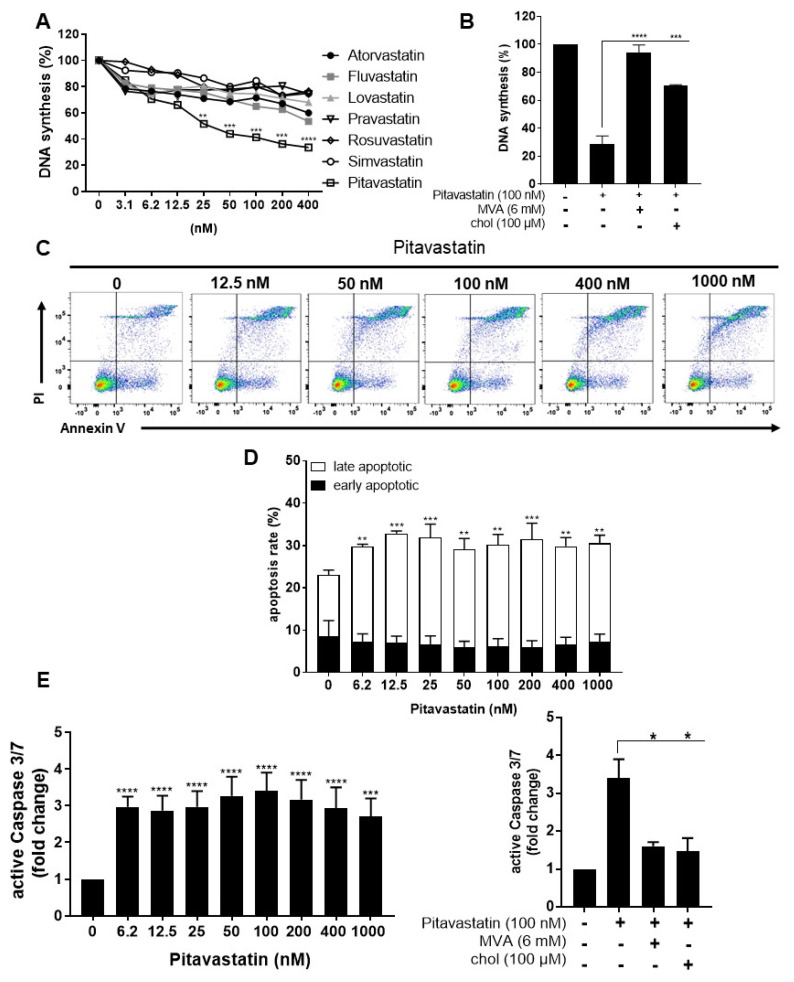
Pitavastatin suppressed proliferation and induced apoptosis in pre-activated T cells. Resting human T cells were pre-activated for 48 h with anti-CD3/CD28 antibodies and cultured with increasing concentrations of different statins (**A**) or 100 nM Pitavastatin in the presence or absence of 6 mM MVA or 100 µM cholesterol (chol) (**B**) for 72 h. DNA synthesis was determined by standard [^3^H]-thymidine uptake. [^3^H]-TdR incorporation is shown as mean percentage of DNA synthesis in relation to control cultures (vehicle) set to 100%. Cells were stained after 72 h with Annexin V-FITC/PI for flow cytometric analysis. (**C**) Representative flow cytometry dot plots and (**D**) quantification of early and late apoptotic cells of *n* = 3 independent experiments are shown. (**E**) Resting human T cells were pre-activated for 48 h with anti-CD3/CD28 antibodies and cultured with increasing concentrations of Pitavastatin (left) or 100 nM Pitavastatin in the presence or absence of 6 mM MVA or 100 µM cholesterol (chol) (right). Cells were stained after 72 h with CellEvent™ Caspase-3/7 Green detection reagent. Relative caspase expression of *n* = 3 independent experiments is presented as mean + SEM. Statistical analysis was performed with One-Way ANOVA and Dunnett’s Multiple Comparison Analysis Test as post hoc test. (**** *p* ≤ 0.0001, *** *p*  ≤  0.001, ** *p * ≤  0.01, * *p*  ≤  0.05).

Activated autoreactive T cells are key players in the immunopathogenesis and development of various autoimmune diseases. Therefore, it is crucial to find compounds that not only prevent activation of naïve resting T cells, but also efficiently suppress already activated T cells. To investigate the effect of Pitavastatin on the proliferation of pre-activated T cells, human T cells were stimulated with anti-CD3/CD28 antibodies for 48 h. After this period, cells were incubated with Atorvastatin, Fluvastatin, Lovastatin, Pravastatin, Rosuvastatin, Simvastatin and Pitavastatin, at concentrations ranging from 3.12 to 400 nM, for an additional 24 h. Proliferation was assessed by measuring [^3^H]-TdR incorporation after 72 h (Figure 6A). Interestingly, only Pitavastatin treatment strongly and dose-dependently suppressed anti-CD3/CD28-mediated proliferation of pre-activated T cells (IC50 value: 48.5 nM). We did not determine an IC50 value for any other statin in the tested concentration range. The Pitavastatin-dependent inhibition of T-cell proliferation was completely restored by the addition of MVA and partially by the addition of cholesterol (Figure 6B). These data indicate that Pitavastatin, in contrast to the other statins, inhibited the proliferation of pre-activated T cells at nanomolar concentrations in a dose-dependent manner. 

In further experiments, pre-activated T cells were incubated with increasing concentrations of Pitavastatin for additional 24 h and subsequently stained with Annexin V-FITC/PI (Figure 6C,D). Flow cytometric analysis revealed a significant increase in the percentage of late apoptotic cells at all Pitavastatin concentrations applied. Thus, in pre-activated T cells treated with 100 nM of Pitavastatin, the percentage of late apoptotic cells increased significantly from 23.1 ± 3.9% up to 30.2 ± 3.4%.

Moreover, we measured the activity of the caspase-3 and -7 as downstream effectors of the apoptotic signaling cascade in pre-activated T cells after treatment with Pitavastatin. As shown in Figure 6E left, Pitavastatin induced activation of caspase-3/7 at all applied concentrations. Active caspase-3/7 was elevated up to 3.4-fold in cells treated with 100 nM Pitavastatin compared to untreated controls. Supplementation of MVA or cholesterol reduced caspase-3/7 activation to a degree observed in control cells (Figure 6E, right). Taken together, these results suggest that Pitavastatin induced apoptosis in pre-activated T cells in a cholesterol-dependent manner.

## 3. Discussion

Statins suppress cholesterol synthesis by inhibiting HMG-CoA reductase activity, thereby depleting MVA, a cholesterol precursor, and consequently cholesterol. However, multiple pleiotropic effects of statins beyond their cholesterol-lowering effects have attracted attention [29]. Thus, in vitro and in vivo studies suggest that statins also cause direct anti-inflammatory [30] and immunomodulatory effects [31]. Previous studies have reported that statins inhibit T-cell proliferation [32,33]. 

In the present study, we analyzed the effect of several statins on the proliferation of freshly stimulated human T cells. Among the statins investigated, Pitavastatin showed the strongest suppression of T-cell proliferation with an IC50 of 3.6 nM. Importantly, inhibition of cell proliferation by Pitavastatin observed in our study was also detected in pre-activated T cells. This is a clear advantage of Pitavastatin as a potential immunosuppressive drug compared to other statins, because activated autoreactive T cells are key players in autoimmune diseases [12]. We previously showed that even some well-established immunosuppressive drugs, such as dexamethasone and cyclosporin A, are not capable of suppressing the proliferation of pre-activated T cells [13]. In our experimental setting, only Pitavastatin suppressed the proliferation of pre-activated T cells, while other statins showed no significant inhibitory effects on T-cell proliferation measured by [^3^H]-thymidine incorporation. Further investigations will focus on structural analysis and structural comparison of Pitavastatin with other statins, as well as with other potential immunosuppressive drugs, e.g., Adefovir Dipivoxil [14].

The immunomodulatory effects of statins are mainly dependent on the HMG-CoA pathway [34]. In our experiments, the inhibition of T-cell proliferation was rescued by addition of MVA in both experimental conditions, confirming that the inhibitory effect was reversible, and a direct result of statin-mediated inhibition of the HMG-CoA pathway. Contrary to many other studies [35,36,37,38], supplementation of FPP or GGPP could not reverse the Pitavastatin-induced proliferation inhibition. This suggests that the inhibition of prenylation is not responsible for the observed effect on T-cell proliferation. In contrast, addition of cholesterol partially reversed the Pitavastatin-induced anti-proliferative effect. It is known that fatty acid biosynthesis, including cholesterol biosynthesis, is strongly upregulated in proliferating T cells [39]. Inhibition of cholesterol synthesis leads to a decrease in DNA synthesis in highly proliferating cell populations, such as lymphocytes or fibroblasts [40,41].

The expression of the early activation marker CD69 is induced by TCR engagement and dependent on Ras activation [42], while CD25 expression is predominantly dependent on NFAT activation [43]. Although we observed Pitavastatin as a strong inhibitor of T-cell proliferation, the substance did not affect the expression of the activation markers CD69 and CD25. This suggests that Pitavastatin has no influence on TCR-induced T-cell activation. Blank et al. could show that treatment of CD4+ T cells with Atorvastatin leads to diminished expression of CD69 and CD25 [32]. Ghittoni et al. also reported that Simvastatin significantly reduced the expression of CD69 and CD25 on PBMCs [44]. However, in these studies, cells were pre-incubated with statins for 24 h, and the used statin concentrations were much higher than in our experiments. These differences in the experimental setup could lead to cholesterol depletion before stimulation and might explain the impaired TCR activation in these studies.

In addition to the inhibition of T-cell proliferation, we detected a significant decrease in IL-17 and IL-10 production in stimulated T cells treated with Pitavastatin. This is in agreement with data from experiments with a mouse model of autoimmune myocarditis [20]. Furthermore, in vitro studies using human Th1 cells showed decreased IL-10 production after statin treatment [45]. We did not detect significant changes in the production of IFN-γ and IL-5. This is in contrast to decreased IFN-γ levels reported by Chen et al. after incubation of PMA-stimulated T cells with 5–10 µM Pitavastatin [26]. The different genetic background of donors and differences in the statin concentrations used likely explain these discrepancies in cytokine responses.

To better understand the mechanism through which Pitavastatin inhibits proliferation, we analyzed the influence of Pitavastatin on cell-cycle phases and apoptosis. Unexpectedly, we found a cell-cycle arrest only in stimulated T cells treated with Pitavastatin concentrations >400 nM. However, we detected an increase in apoptosis of freshly stimulated T cells at all applied Pitavastatin concentrations. These Pitavastatin-mediated effects were also observed in pre-activated T cells, implicating the same mechanism in both experimental conditions. Previous studies showed that statins induce cell-cycle arrest of human and mouse T cells by an increased protein expression of p27^kip1^ and induction of p21 [46,47]. This effect of statins was also described for other cell types, including tumor cells [48,49,50]. 

Samson et al. and Brinkkoetter et al. reported that statins are capable of inducing apoptosis in human CD4+ T cells [46,51]. Moreover, it has been demonstrated that statins induce cell death in various cancer cells, such as lymphoma cells [52], pancreatic cancer cells [53], cholangiocarcinoma cells [27] and glioblastoma cell lines [54]. The data of our experiments suggest that the observed inhibition of T-cell proliferation by Pitavastatin was due most probably to increased apoptosis caused by lack of MVA. Apoptosis is induced by various stimuli. Depending on these stimuli, two major pathways of apoptosis exist: the extrinsic, or death-receptor, pathway, which involves caspase-8; and the intrinsic or mitochondrial pathway, which can trigger the release of cytochrome c and lead to activation of caspase-9 and downstream caspases (caspase-3 or -7) [55,56]. We observed that Pitavastatin increased the expression of active caspase-8, -9 and -3/7 in freshly stimulated T cells. Based on these findings, we suggest that Pitavastatin might mediate both the intrinsic and extrinsic pathways of apoptosis. Similar findings were reported for human CD4+ T cells [51] and hepatocytes [57] in which statin treatment activated caspase-8, -9, and -3. Activation of caspase-9 and caspase-3/7 by statins was also observed in glioblastoma, non-small lung cancer cells and breast cancer cell lines [58]. In our experiments, supplementation of MVA was able to suppress the activity of caspase-9 and -3/7 induced by Pitavastatin, implicating that blockade of the MVA pathway triggers the cascade of the intrinsic apoptosis pathway. Furthermore, cholesterol was capable of significantly inhibiting Pitavastatin-induced activation of caspase-9 and -3/7. These data support the hypothesis that Pitavastatin-induced apoptosis is due to a depletion of MVA and cholesterol. Kinetic analysis of caspase-3/7 activation showed an increase in caspase activity at later timepoints, indicating a delayed apoptosis induction. This is in line with findings of Kuzyk et al., showing Simvastatin-induced delayed apoptosis in neuroblastoma cells [59]. Of note, we found the highest apoptosis induction in T cells treated with Pitavastatin concentrations <1 µM. At higher Pitavastatin concentrations, apoptosis was attenuated, implicating a biphasic effect of this drug. Concentration-dependent effects of Pitavastatin have been observed already by other authors. Wang et al. showed that Pitavastatin at a low dose activated eNOS in endothelial cells, while higher concentrations inhibited eNOS [60]. Furthermore, Pitavastatin enhanced the migration, proliferation and viability of vascular endothelial cells at low concentrations, but it inhibited these cellular functions at high concentrations [61]. In future investigations, this biphasic effect of Pitavastatin should be explored in more detail in animal models of T-cell-mediated autoimmune diseases, e.g., EAE as model of multiple sclerosis in vivo.

The Ras/Raf/extracellular signal-regulated kinase (ERK) signaling pathway plays a crucial role in various cellular functions such as proliferation and cell survival [62]. Ghittoni et al. reported that Simvastatin inhibited activation of ERK1/2 in T cells [44]. Statin-induced inhibition of ERK1/2 activation was observed in T cells, as well as in other cell types, such as glioblastoma cells and cholangiocarcinoma cell lines [26,27,28]. However, in our study, we found a sustained phosphorylation of ERK1/2 in human T cells upon Pitavastatin treatment. This effect was clearly visible after 72 h upon stimulation, thus suggesting that Pitavastatin regulates ERK1/2 activation independently of the initial TCR signal. Similar to apoptosis induction, we observed the strongest ERK1/2 phosphorylation in T cells treated with lower Pitavastatin concentrations (<1 µM), while high concentrations inhibit ERK1/2 phosphorylation. Because of this observed pattern, we hypothesized that sustained ERK1/2 phosphorylation is responsible for Pitavastatin-induced apoptosis in T cells. The apoptosis induction in T cells treated with Pitavastatin was clearly dependent on the ERK1/2 phosphorylation as a consequence of the Ras/Raf/MEK cascade activation, because treatment with the MEK inhibitor U0126 reverted this effect. Although ERK1/2 is a pro-survival factor of the MAP kinase family, a growing number of studies also suggest that, under certain conditions, ERK activation can promote cell death [63]. The pro-apoptotic function of ERK1/2 is known for apoptosis induced by antitumor compounds, such as Quercetin [64] or Taxol [65]. Depending on the cell type and stimulus, ERK activation is either associated with the intrinsic apoptosis pathway [66] or with the extrinsic apoptosis pathway [67]. ERK activity has been shown to directly affect mitochondrial function by decreasing mitochondrial membrane potential leading to mitochondrial membrane disruption, cytochrome c release and subsequently to activation of caspase-9 and -3 [68]. ERK1/2 activity can directly influence the expression of pro-apoptotic proteins, such as Bax [69] and Bak [70]. Li et al. showed that ERK1/2-activated caspase-8 induced the release of cytochrome c through proteolytic activation of the pro-apoptotic molecule Bid [71]. 

Supplementation with MVA and cholesterol could prevent Pitavastatin-induced ERK1/2 hyperphosphorylation, implicating a cholesterol-dependent effect. Cholesterol can modulate T-cell activation via changing dynamics of lipid rafts thereby altering the localization of important signaling molecules. It is well-known that functions of lipid rafts are dependent on cholesterol concentration [72,73]. Zhuang et al. showed that Simvastatin lowered the cholesterol content in lipid rafts and subsequently altered signaling and induced apoptosis [74]. Of note, Furuchi and Anderson reported that depletion of membrane cholesterol dramatically induced phosphorylation of ERK1/2 in the cytosol [75]. 

## 4. Materials and Methods

### 4.1. Reagents

Atorvastatin, Fluvastatin, Lovastatin, Pravastatin, Rosuvastatin and Simvastatin were purchased from Enzo Life Sciences, Inc. (Farmingdale, NY, USA); Pitavastatin, cholesterol and U0126 from Selleckchem (Houston, USA); Farnesyl Pyrophosphate (FPP) and Geranylgeranyl Pyrophosphate (GGPP) from Biomol GmbH (Hamburg, Germany); and Mevalonic acid lactone (MVA) from Sigma Aldrich (St. Louis, MO, USA). Hybridoma supernatants containing mouse anti-human CD3ε (OKT-3) and CD28 (248.23.2) monoclonal antibodies were produced and purified in our laboratory.

### 4.2. Cells

Human PBMCs were isolated by density gradient (Biochrom, Berlin, Germany) centrifugation of heparinized blood collected from 39 healthy volunteers (24 women and 15 men; age range 21–69 years; mean age 34.9 years). Human T cells were further purified by non-T-cell depletion, using the “Pan T cell isolation kit II” (Miltenyi Biotec GmbH, Bergisch Gladbach, Germany). The resulting pan T-cell purity was >97%. Cells were washed twice and resuspended in serum-free AIM-V culture medium (Invitrogen, Eggenstein, Germany). The study was approved by the local ethics committee (No. 141/19). All blood donors gave their written informed consent.

### 4.3. T-Cell Proliferation Assay

Resting human T cells (10^5^ cells/100 μL) were incubated in quadruplicate cultures in 96-well microtiter culture plates (TPP Techno Plastic Products AG, Trasadingen, Switzerland) coated with anti-CD3e (OKT-3) and anti-CD28 (248.23.2) monoclonal antibodies. To investigate the effect on freshly stimulated T cells, increasing concentrations of statins and 6 mM MVA, 100 µM cholesterol, 10 µM FPP or 10 µM GGPP were directly added as indicated. To study the effect on pre-activated T cells, compounds were added 48 h after stimulation. All cell cultures were incubated for 72 h at 37 °C. Proliferation was assessed by measuring [^3^H]-thymidine ([3H]-TdR) incorporation. Therefore, [^3^H]-TdR (Perkin Elmer, Boston, MA, USA) was added at 0.2 μCi/well for the last 6 h of the incubation. At the end of the incubation period, cells were harvested, and radioisotope incorporation was measured using the betaplate liquid scintillation counter MicroBeta (Wallac, Turku, Finland). Results of at least four independent experiments were calculated as mean percentage ± Standard error of the mean (SEM) of DNA synthesis in relation to control cultures set to 100%.

### 4.4. Flow Cytometric Analysis of Activation Markers

Activated T cells were treated with various concentrations of Pitavastatin or vehicle control for 16 h (CD69) or 48 h (CD25). Afterwards the cells were transferred to polystyrene tubes, washed with FACS staining buffer (BioLegend, San Diego, CA, USA) and incubated with allophycocyanin (APC)-labeled anti-CD69 antibodies or phycoerythrin (PE)-labeled anti-CD25 antibodies, at 4 °C, for 30 min. At the end of the incubation period, cells were washed with phosphate-buffered saline (PBS)/2 mM ethylenediaminetetraacetic acid (EDTA), resuspended in FACS staining buffer and analyzed by flow cytometry (LSRFortessa, BD Biosciences, Franklin Lakes, NJ, USA). Data of at least four independent experiments were acquired by FACSDiva 6.0 software (BD Biosciences) and analyzed by using the FlowJo software (version 7.6.4, Tree Star Inc., Ashland, OR, USA).

### 4.5. Cytokine ELISA

For measurement of cytokine secretion, human T cells (10^6^ cells/mL) were seeded in anti-CD3/CD28 antibody-coated 24-well plates (Corning Inc., Amsterdam, The Netherlands) and treated with increasing concentrations of Pitavastatin or vehicle control. Supernatants were harvested after 72 h, and cytokine concentrations (IFN-γ, IL-5, IL-10 and IL-17) were determined with specific ELISA (bio-techne Ltd., Minneapolis, MN, USA), according to manufacturer’s instructions. The optical density of each well was determined at 450 and 570 nm (Tecan Safire Microplate Reader, Tecan Group, Männedorf, Switzerland). The concentration of each cytokine was calculated by using a standard curve and the measured absorbance (*n* = 4 independent experiments).

### 4.6. Apoptosis measurement

Cell apoptosis was detected by using the Annexin V-FITC Apoptosis Detection Kit with propidium iodide (PI) (BioLegend). Briefly, cells were washed with PBS and re-suspended in 1x binding buffer. Then cells (0.5 × 10^6^/100 µL) were incubated with 2.5 μL of Annexin V-FITC and 2.5 μL of PI for 15 min, at room temperature, in the dark. The incubation was terminated by the addition of 200 μL of 1x binding buffer. Samples were analyzed by flow cytometry, using LSRFortessa (BD Biosciences, Franklin Lakes, NJ, USA). Early apoptotic cells were defined by Annexin-V-positive and PI-negative staining. Late apoptotic and non-viable cells were identified based on both Annexin-V- and PI-positive staining. At least 20,000 cells were examined for each sample. The data of at least four independent experiments were analyzed by using the FlowJo software (version 7.6.4, Treestar Inc., Ashland, OR, USA).

### 4.7. Flow Cytometric Analysis of Active Caspase-3/7 

Caspase-3 and caspase-7 activation was determined by using the CellEvent™ Caspase-3/7 Green Flow Cytometry Assay Kit (Thermo Fisher Scientific, Waltham, MA, USA). The membrane-permeable substrate is cleaved by activated caspase-3 or caspase-7 in apoptotic cells, enabling the dye to bind to DNA and thereby inducing a green fluorescent signal. Cells were incubated with 500 nM CellEvent™ caspase-3/7 green detection reagent in PBS for 30 min, at room temperature, in the dark. Quantitative analysis of 20,000 cells per sample was performed on a flow cytometer (FACS Calibur, BD Biosciences, Franklin Lakes, NJ, USA) and analyzed by using the FlowJo software (*n* = 3 independent experiments).

### 4.8. IncuCyte Caspase-3/7 Assay

An IncuCyte S3 Live-Cell Imaging system (Essen Bioscience, Ann Arbor, MI, USA) was used for kinetic monitoring of apoptotic activity of Pitavastatin in T cells. Therefore, T cells were seeded in quadruplicate cultures in 96-well plates (at a density of 3 × 10^4^ cells/well), and Pitavastatin or vehicle control was added in various concentrations. For fluorescence-based cell-death analysis, IncuCyte™ Caspase-3/7 Green Reagent (Essen Bioscience, 1.6 µM) was added. Thereafter, plates were placed into the IncuCyte S3 Live-Cell Analysis System at 37 °C. Every 3 h, images of each well were taken at 10× magnification, over a time period of 84 h. Images of green fluorescence were monitored and automatically quantified by the IncuCyte S3 v2018B software (Essen Bioscience, Ann Arbor, MI, USA). Images were analyzed concerning numbers of green objects per well (*n* = 3 independent experiments).

### 4.9. Flow Cytometric Caspase-8 and Caspase-9 Assay

Cells were analyzed by using CaspGLOW Fluorescein Active Caspase-8 Staining Kit and CaspGLOW Fluorescein Active Caspase-9 Staining Kit (Invitrogen, Carlsbad, CA, USA). The caspase-8 inhibitor IETD-fluoromethylketone conjugated to FITC (FITC-IETD-FMK), and caspase-9 inhibitor LEHD-fluoromethylketone conjugated to FITC (FITC-LEHD-FMK) were irreversibly bound to active caspase-8 or active caspase-9 in apoptotic cells, respectively. Briefly, T cells in the presence of Pitavastatin or vehicle controls were centrifuged for 5 min at 400× *g*, and the supernatant was removed by aspiration. The cell pellet was resuspended in 200 μL of PBS, containing FITC-IETD-FMK or FITC-LEHD-FMK at a dilution of 1:500, and the cells were incubated for 30 min at 37 °C. Afterwards, the cell suspension was centrifuged, the supernatant was removed and the cells were washed twice with 300 μL wash buffer. After the final washing step, cells were resuspended in 200 μL wash buffer and analyzed by using flow cytometry (FACS Calibur, BD Biosciences, Franklin Lakes, NJ, USA). The data of at least three independent experiments were analyzed by using the FlowJo software.

### 4.10. Western Blot

Cells were lysed by using radioimmunoprecipitation assay buffer (RIPA)-based lysis buffer supplemented with Na_3_VO_4_, Phenylmethylsulfonylfluorid (PMSF) and complete EDTA-free protease inhibitor mixture (Roche, Basel, Switzerland). Protein concentrations were determined by Pierce BCA Protein Assay Kit (Thermo Fisher Scientific, Waltham, MA, USA). Equal amounts of protein were separated on sodium dodecyl sulfate (SDS) polyacrylamide gels and transferred onto nitrocellulose membrane. Membranes were blocked with 5% non-fat dry milk in Tris-buffered saline (TBS) containing 0.1% Tween-20 (TBS-T) for 1 h. After blocking, the membrane was incubated with the primary antibody, overnight, at 4 °C. Next, the membrane was washed three times with TBS-T and incubated with the appropriate secondary antibody, at a dilution of 1:10,000, for 1 h, at room temperature (LI-COR Biosciences, Lincoln, NE, USA). The immunoreactive bands were visualized by using an Odyssey infra-red scanner (LI-COR Biosciences, Lincoln, NE, USA). The following primary antibodies were used: rabbit anti-P-Erk1/2 antibody and mouse anti-p44/42 MAP Kinase antibody (Cell Signaling Technology, Danvers, MA, USA).

### 4.11. Cell-Cycle Analysis

Cells treated with Pitavastatin at varying concentrations were centrifuged at 200× *g* for 5 min, at room temperature. After aspirating the medium, cells were resuspended in cold PBS/1% BSA. The cells were then fixed in 70% ethanol, while vortexing, and stored at −20 °C for 2 h. Before analysis, cells were washed two times at 200× *g*, for 10 min, at room temperature, to remove the ethanol. Cells were rinsed twice with PBS/1% BSA and stained for 15 min with 1 μg/mL PI/RNase staining solution (BD Biosciences, Franklin Lakes, NJ, USA). The samples were measured by FACSCalibur (BD Biosciences). The percentage of cells at each cell-cycle phase was quantified by fitting the experimentally obtained histogram with the Dean–Jett–Fox model in FlowJo software version 7.6.4 software (Treestar Inc., Ashland, OR, USA) (*n* = 4 independent experiments).

### 4.12. Statistical Analysis

GraphPad Prism software version 7.0 (Graph Pad Software, La Jolla, CA, USA) was used for statistical analysis. Significance levels were calculated by repeated measures of One-Way ANOVA and Dunnett’s Multiple Comparison Analysis Test as post hoc test with 95% confidence interval (α = 0.05). Data were presented as the mean with standard error of the mean (mean ± SEM). Within figures, the P-values are displayed with asterisks (**** *p* ≤ 0.0001, *** *p*  ≤  0.001, ** *p*  ≤  0.01, * *p*  ≤  0.05). The IC50 values were calculated by nonlinear least-squares regression analysis with GraphPad Prism software version 7.0, using Prism’s non-linear regression dialog (curvefit).

## 5. Conclusions

**Figure 7:**
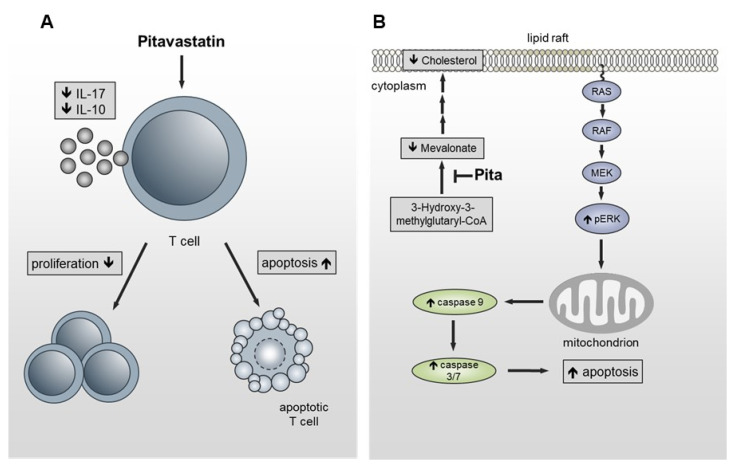
Schematic presentation of the functional effects of Pitavastatin on T cells. (**A**) Pitavastatin inhibits proliferation in freshly stimulated and pre-activated T cells. Inhibition of proliferation was associated with reduced IL-17 and IL-10 secretion and apoptosis induction. (**B**) By inhibiting HMG-CoA reductase, the cholesterol concentration in the lipid rafts is reduced, leading to hyperphosphorylation of ERK1/2. Sustained activation of ERK1/2 induces the activation of caspase-9 and caspase-3/7, leading to cell death.

In summary, we identified Pitavastatin as being a very potent FDA-approved compound with a strong anti-proliferative effect on freshly stimulated, as well as on pre-activated human T cells at low nanomolar concentrations. The inhibition of T-cell proliferation is most probably due to increased apoptosis. By inhibiting HMG-CoA reductase, the cholesterol concentration in the membrane is reduced, leading to the hyperphosphorylation of ERK1/2. Sustained activation of ERK1/2 induced, in turn, the activation of caspase-9 and caspase-3/7, thus leading to cell death (Figure 7). Due to the fact that Pitavastatin has a more favorable pharmacological profile compared to other statins [76] and safety and efficacy has been proven in several studies, it represents a new potential candidate for low-dose treatment of T-cell-mediated autoimmune diseases. Further in vivo investigations, as well as preclinical and translational studies, should prove the option of immunosuppressive therapy with Pitavastatin in autoimmune diseases. Subsequently, prospective clinical studies should be performed to test the possible therapeutic application of Pitavastatin for such diseases.

## Data Availability

The data presented in this study are contained within the article.
